# Apigenin as Tumor Suppressor in Cancers: Biotherapeutic Activity, Nanodelivery, and Mechanisms With Emphasis on Pancreatic Cancer

**DOI:** 10.3389/fchem.2020.00829

**Published:** 2020-10-15

**Authors:** Milad Ashrafizadeh, Mohammad Reza Bakhoda, Zahra Bahmanpour, Khandan Ilkhani, Ali Zarrabi, Pooyan Makvandi, Haroon Khan, Samaneh Mazaheri, Maryam Darvish, Hamed Mirzaei

**Affiliations:** ^1^Department of Basic Science, Faculty of Veterinary Medicine, University of Tabriz, Tabriz, Iran; ^2^Student Research Committee, Shahrekord University of Medical Sciences, Shahrekord, Iran; ^3^Department of Medical Genetics, Faculty of Medicine, Tabriz University of Medical Sciences, Tabriz, Iran; ^4^Sabanci University Nanotechnology Research and Application Center (SUNUM), Istanbul, Turkey; ^5^Centre for Micro-BioRobotics, Istituto Italiano di Tecnologia, Pisa, Italy; ^6^Department of Medical Nanotechnology, Faculty of Advanced Technologies in Medicine, Iran University of Medical Sciences, Tehran, Iran; ^7^Department of Pharmacy, Abdul Wali Khan University Mardan, Mardan, Pakistan; ^8^Department of Analytical Chemistry, Faculty of Chemistry, University of Kashan, Kashan, Iran; ^9^Department of Medical Biotechnology, Faculty of Medicine, Arak University of Medical Science, Arak, Iran; ^10^Research Center for Biochemistry and Nutrition in Metabolic Diseases, Institute for Basic Sciences, Kashan University of Medical Sciences, Kashan, Iran

**Keywords:** apigenin, pancreatic cancer, oxidative stress, apoptosis, therapy

## Abstract

Pancreatic cancer is the most lethal malignancy of the gastrointestinal tract. Due to its propensity for early local and distant spread, affected patients possess extremely poor prognosis. Currently applied treatments are not effective enough to eradicate all cancer cells, and minimize their migration. Besides, these treatments are associated with adverse effects on normal cells and organs. These therapies are not able to increase the overall survival rate of patients; hence, finding novel adjuvants or alternatives is so essential. Up to now, medicinal herbs were utilized for therapeutic goals. Herbal-based medicine, as traditional biotherapeutics, were employed for cancer treatment. Of them, apigenin, as a bioactive flavonoid that possesses numerous biological properties (e.g., anti-inflammatory and anti-oxidant effects), has shown substantial anticancer activity. It seems that apigenin is capable of suppressing the proliferation of cancer cells *via* the induction of cell cycle arrest and apoptosis. Besides, apigenin inhibits metastasis via down-regulation of matrix metalloproteinases and the Akt signaling pathway. In pancreatic cancer cells, apigenin sensitizes cells in chemotherapy, and affects molecular pathways such as the hypoxia inducible factor (HIF), vascular endothelial growth factor (VEGF), and glucose transporter-1 (GLUT-1). Herein, the biotherapeutic activity of apigenin and its mechanisms toward cancer cells are presented in the current review to shed some light on anti-tumor activity of apigenin in different cancers, with an emphasis on pancreatic cancer.

## Introduction

### Pancreatic Cancer: A Brief Representation

Pancreatic cancer (PC) is the fourth leading cause of malignancy-associated mortality with <5% 5-years survival. Clinical strategies for the management of this cancer have recently been developed; however, the mortality rate is still mostly unaltered (Siegel et al., 2018). This high rate of mortality is attributed to the aggressive nature of this cancer as well as a lack of efficient therapy methods (Tempero et al., 2017). The high metastatic ability of PC cells, and also, their uncontrolled growth have led to some difficulties in the effective treatment of this life-threatening disorder.

Although chemotherapy and surgery are the most common methods in cancer therapy, growing evidence demonstrates that the aforementioned strategies are only effective in a few numbers of patients. Consequently, radiotherapy is also used to enhance the efficacy of chemotherapy. However, it seems that combination chemotherapy with other anti-tumor agents would be the best strategy for cancer treatment. This is due to the fact that cancer cells are able to acquire resistance toward both radiotherapy and chemotherapy; accordingly, combination chemotherapy facilitates the disruption molecular pathways involved in cancer resistance. Subsequently, the effectiveness of chemotherapy is ameliorated and its clinical trial findings would be more satisfactory. Finding a suitable anti-tumor agent in combination with chemotherapy is of importance in poly-chemotherapy. High anti-tumor activity, multi-targeting, and minimal toxicity are some of the most important properties of an ideal anti-tumor agent (Lee et al., 2019b,c, 2020; Tan and Norhaizan, 2019; Banik et al., 2020; Patra et al., 2020).

To date, a wide variety of strategies were employed in suppressing chemoresistance, and malignant behavior of PC cells. Among them, plant derived-natural products are of importance in PC due to their excellent anti-tumor activity, and capability of enhancing sensitivity in PC cells into chemotherapy (Cheng et al., 2018; Yan et al., 2018). In light of this, much attention was directed toward using plant derived-natural compounds as potential anti-tumor agents for use in combination chemotherapy, and for suppressing malignant behavior and the proliferation of cancer cells (Abotaleb et al., 2020; Liskova et al., 2020; Varghese et al., 2020). In respect to the fact that a variety of molecular pathways are involved in the progression and proliferation of PC cells such as Wnt (Xu et al., 2020), Nrf 2 (Krajka-Kuzniak et al., 2020), long non-coding RNAs (lncRNAs) (Yin et al., 2020), and microRNAs (miRs) (Wang et al., 2020) its effective therapy relies on using anti-tumor compounds with the capability of the induction of onco-suppressor pathways, and the inhibition of oncogene ones. Notably, naturally occurring compounds are capable of modulating molecular pathways and mechanisms. It seems that Akt is an oncogene pathway involved in the proliferation and viability of PC cells. The administration of curcumin, as a naturally occurring nutraceutical compounds, remarkably reduces Akt expression by suppressing its upstream modulator epidermal growth factor (EGF), leading to a decrease in growth and malignant behavior of the PC cells (Li et al., 2019). The curcumin analogs have demonstrated more inhibitory effects on the proliferation of pancreatic cancer cells due to their enhanced bioavailability (Nagaraju et al., 2019). Besides, phytochemicals are able to interfere with metastasis and the invasion of PC cells by suppressing epithelial-to-mesenchymal transition (EMT) (Hoca et al., 2019). It is worth mentioning that plant derived-natural compounds are beneficial in enhancing the sensitivity of PC in chemotherapy (Zhou et al., 2019). These studies are in line with the potentiality of herbal-based products in PC therapy. Herein, we aim to explore the anti-tumor activity of apigenin, as a natural compound, on different cancers with a special focus on PC.

### Apigenin: Chemical Structure and Biological Functions

Apigenin (4′,5,7-trihydroxyflavone) is a plant-derived material belonging to the flavone category that is the aglycone of several naturally occurring glycosides. The molecular formula and molecular weight of apigenin are C_15_H_10_O_5_ and ~270 g/mol, respectively. Flavones and several of their synthetic derivatives are well-known for their biological and therapeutic activities, including anti-oxidant, anti-inflammatory, anti-cancer, ant-genotoxic, anti-allergic, neuroprotective, cardioprotective, and antimicrobial (Catarino et al., 2015). Apigenin is a yellow crystalline solid and its main non-pharmaceutical application is its use to dye wool. Compared to other structurally related flavonoids, apigenin was a useful and health promoting agent in recent years due to its low toxicity and significant effects on normal vs. cancer cells (Gupta et al., 2001). The solid therapeutic potential of apigenin against various diseases was proven through evidence achieved by numerous studies. The prior art has not been able to provide a robust proof to indicate that apigenin increases the negative metabolic responses *in vivo* when consumed as part of a normal diet. However, the results of some investigations in Swiss mice proposed the oxidative stress-induced liver damage, which may be due to the stimulation of multiple genes *via* apigenin at higher doses (Singh et al., 2012). The strong anti-oxidant and anti-inflammatory activities of apigenin are a substantial reason for its possible cancer preventive effects (Singh et al., 2012). Encouraging metal chelation, scavenging free radicals, and triggering phase II detoxification enzymes in cell cultures as well as *in vivo* tumor models are also functions of apigenin (Middleton et al., 2000). More importantly, apigenin significantly contributes in the prevention of cancer by inducing apoptosis in different cell lines as well as animal models (Kaur et al., 2008).

### Pharmacokinetics of Apigenin: A Brief Explanation

Owing to outstanding pharmacological activities of apigenin, a number of studies have exploited the pharmacokinetics of apigenin to demonstrate its absorption, metabolism, distribution, and excretion. Such findings are beneficial for directing further studies to use an optimal dose of apigenin in disease therapy (Wang et al., 2019a). It was reported that after the consumption of polyphenols, 5–10% of apigenin may be absorbed (Cardona et al., 2013). The gastrointestinal tract (GIT) is involved in the absorption of apigenin before its arrival in blood circulation and the liver. Upon aglycone apigenin administration, its immediate absorption occurs in the intestine (based on a perfused rat intestinal model) (Liu and Hu, 2002). It is worth mentioning that different parts of the intestine have various absorption routes for apigenin. For instance, passive and active carrier-mediated saturable mechanisms contribute to the absorption of apigenin in the duodenum and jejunum, while its absorption occurs in the ileum and colon *via* passive transportation (Zhang et al., 2012). However, there are conflicting data about the rate of apigenin absorption. Although one study is in line with the fact that apigenin has a low absorption rate after oral administration (appearing in blood circulation after 24 h) (Gradolatto et al., 2005), another research confirms its high absorption rate (appearing in blood circulation after 3.9 h) (Chen et al., 2007). Consequently, more studies should be conducted to show the absorption rate of apigenin. In terms of distribution, various studies were performed and it was reported that apigenin is distributed in different organs of the body including the kidney, intestine, and liver. Moreover, half of apigenin intake appeared in urine and feces (Liu and Hu, 2002; Gradolatto et al., 2005; Cai et al., 2007; Wan et al., 2007).

Increasing evidence demonstrates that the metabolism of apigenin consists of two major phases. The phase I metabolism of apigenin occurs in the liver, and at the presence of liver enzymes such as cytochrome P450 with collaboration of nicotinamide adenine dinucleotide phosphate (NADPH) and flavin-containing monooxygenase (FMO) (Cardona et al., 2013; Tang et al., 2017). Enteric and enterohepatic cycling participate in the biotransformation of apigenin in phase II metabolism (Chen et al., 2007). Glucuronidation and sulfation are essential for phase II metabolism (Tang et al., 2017). During metabolism, apigenin is bio-transformed into metabolites including luteolin (Lut) and sulfated and glucuronidated conjugates (Chen et al., 2003; Gradolatto et al., 2005). Regarding the excretion, apigenin appeared in both urine and feces with more concentration in urine. The age and sex of rats are crucial factors that affect the excretion of apigenin. Furthermore, it was shown that metabolism and the excretion of apigenin occur in a slow process, confirming accumulation of apigenin in the body (Gradolatto et al., 2005).

### Chemistry of Apigenin: An Overview

**Figure 1:**
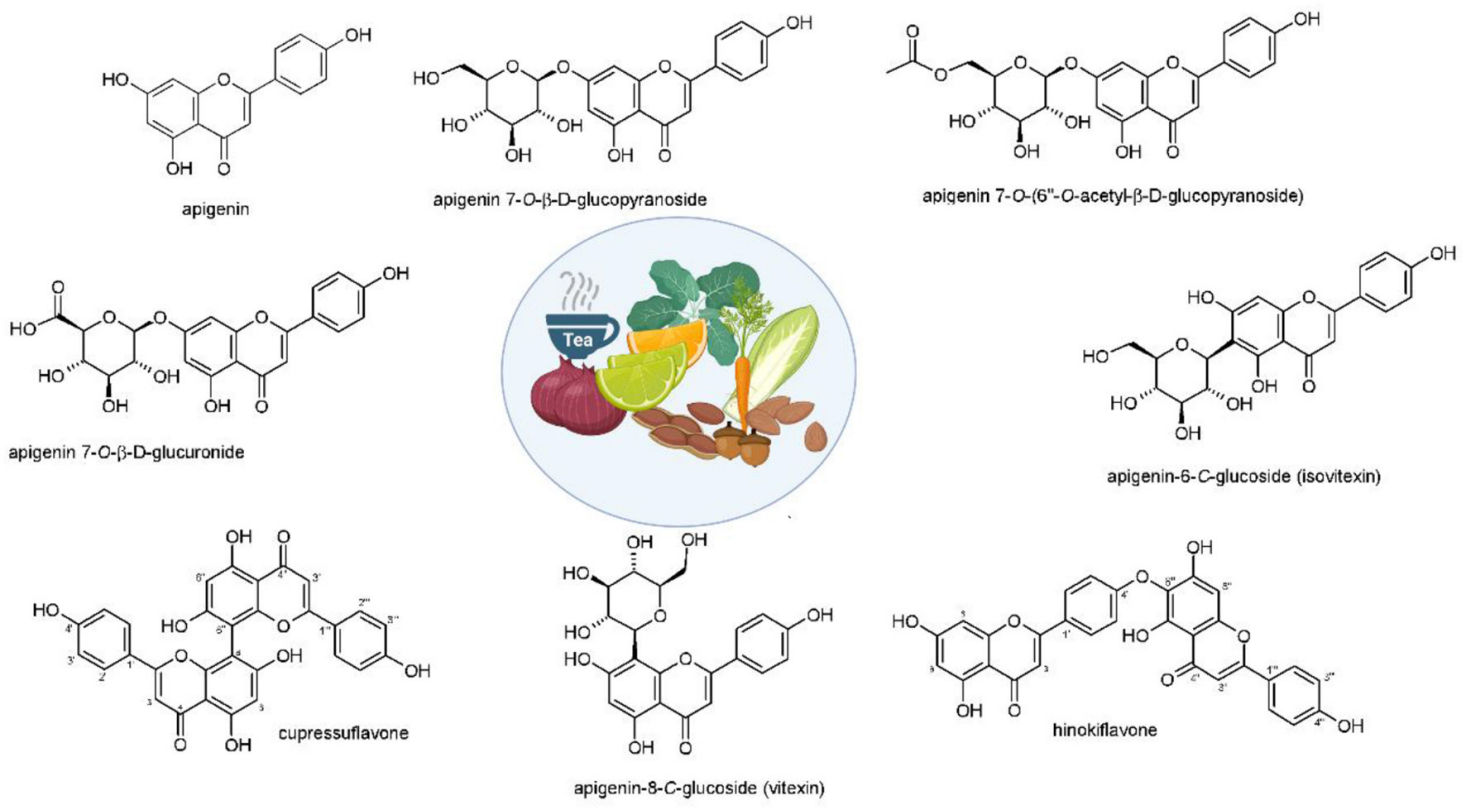
Structures and sources of apigenin and its glycosidic, glucuronide, acetylated, and methyl ester derivatives. Reprinted with modification from Salehi et al. (2019).

Apigenin and its derivatives are found in several sorts of plants, e.g., fruits, vegetables, nuts, citrus, tea, chamomile, thyme, celery, and celeriac in its glycoside form (Figure 1) (Yan et al., 2017; Wang et al., 2019b). Among glycoside forms, apigenin-7-O-glucoside is the major one. In terms of solubility, apigenin is not soluble in water and non-polar solvents (e.g., silicon fluid), while it is soluble in organic solvents such as dimethylsulfoxide (DMSO) (Li et al., 1997; Zhang et al., 2012; Lakshmanan et al., 2015; Wang et al., 2017). It was demonstrated that glycoside and acylated derivatives of apigenin have more solubility in water, compared to apigenin (Shukla and Gupta, 2010). Such changes in the hydrophilicity of apigenin affects its absorption and bioavailability. It was shown that when apigenin is attached into β-glycosides, it possesses the highest bioavailability among other forms (Patel et al., 2007). The excellent therapeutic and biological activities of apigenin are due to the presence of glucosides that promote the stability of apigenin (Gurung et al., 2013). Regarding its storage, it is recommended that apigenin be stored at −20°C, since it is unstable at room temperature (Patel et al., 2007). It is worth mentioning that the degradation of natural compounds rely on the presence of special structures. For instance, hydroxyl groups enhance degradation, while sugar moiety and hydroxyl groups reduce the rate of degradation (Biesaga, 2011).

In respect to the poor bioavailability of apigenin resulted from its slow absorption and metabolism, several studies were conducted on developing nanocarriers for enhancing its bioavailability and improving its therapeutic effects. To date, various types of nanoparticles including liposomes and polymeric nanoparticles were developed for the delivery of apigenin (Karim et al., 2017; Pápay et al., 2017a,b; Telange et al., 2017; Alshehri et al., 2019). Notably, they were able to remarkably enhance therapeutic effects of apigenin and, accordingly, its ability in the treatment of different disorders. In section Apigenin-loaded nanovehicles, we specifically discuss role of nanoparticles in promoting anti-tumor activity of apigenin against cancer cells along with enhancing the efficacy of chemotherapy.

## Apigenin for Cancer Therapy

In addition to anti-inflammatory and anti-oxidant effects, apigenin possesses a significant anti-cancer property in different types of cancer cells, such as breast cancer (Perrott et al., 2017), liver cancer (Qin et al., 2016), PC (Johnson and de Mejia, 2013), prostate cancer (Shukla et al., 2014a), lung cancer (Pan et al., 2013), and colon cancer (Lee et al., 2014). In this section, we provide discussions about the anti-tumor activity of apigenin, and its efficacy in negatively affecting both the proliferation and migration of cancer cells.

Carcinogenesis is known as a multistage procedure that is accompanied by a series of genetic and epigenetic alterations, resulting in the initiation, promotion, and development of cancer (Farhood et al., 2019; Mortezaee et al., 2019a; Woo et al., 2019). Cancer treatment strategies include eradicating tumor cells by stimulating cell apoptosis or preventing cancer cell proliferation by inducing cell cycle arrest (Mortezaee et al., 2019b,c,d). With these remedies, cancer turns into a chronic disease and the survival of patients can be prolonged. Encouraging apoptosis or autophagy, regulating cell cycle, preventing tumor cell migration and invasion, and triggering the patient's immune response were suggested as current strategies (Najafi et al., 2018, 2019a,b; Hashemi Goradel et al., 2019). So far, all of these antitumor activities of apigenin within diverse types of tumors were reported *in vitro* and *in vivo* models. Table 1 represents an overview of anti-cancer features of apigenin and the involved signaling pathways.

**Table 1:** Selected *in vitro* and *in vivo* studies on the therapeutic effects of apigenin in various cancers.

**Cancer**	**Dose (s)**	**Target gene**	**Model**	**Type of cell line**	**Effect (s)**	**References**
Cervical cancer	40 μM	CK2α	*In vitro*	HeLa	Inhibits cell self-renewal capacity	Liu et al., 2015a
Head and neck squamous cell carcinoma	40 μM	CD44, NANOG, and CD105	*In vitro*	HSC-3, HN-8, and HN- 30	Inhibits the expression of cancer stem cell marker	Ketkaew et al., 2017
Osteosarcoma	50 μg/m	Wnt/β-catenin	*In vitro*	U2OS and MG63	Inhibits proliferation and invasion	Liu et al., 2015b
Mesothelioma	50 μM, 20 mg/kg	AKT and c-Jun phosphorylation/ NF-κB	*In vitro, in vivo*	Malignant mesothelioma (MM) cells	Induces Apoptosis	Masuelli et al., 2017
Oral squamous cell carcinoma	100 μM	cyclin D-1 and E	*In vitro*	SCC-25, HaCaT	Inhibits proliferation; Induces apoptosis	Maggioni et al., 2013
Papillary thyroid carcinoma	25 μM	Cdc25C	*In vitro*	BCPAP	Cell cycle arrest and autophagy induction	Zhang et al., 2015
Adenoid cystic carcinoma	40 μM	GLUT-1	*In vitro*	ACC-2	Inhibits proliferation; Induces apoptosis	Fang et al., 2015
Renal cell carcinoma	20 μM, 30 mg/kg	p53	*In vitro, in vivo*	ACHN, 786-0, and Caki-1	Induces cell cycle arrest	Meng et al., 2017
Glioblastoma	25 μM	c-Met	*In vitro*	U87MG and U373MG	Inhibits self-renewal capacity	Kim et al., 2016
	50 μM	TGF-b1	*In vitro*	GL-15	Inhibits angiogenesis	Freitas et al., 2011
Ovarian cancer	20,40 μM	FAK	*In vitro*	A2780	Inhibits adhesion, migration, and invasion	Hu et al., 2008
	20,40 μM	CK2α	*In vitro*	SKOV3	Inhibits the self-renewal capacity	Tang et al., 2015
Leukemia	60 μM	caspase-9 and caspase-3	*In vitro*	HL60	Induces apoptosis	Wang et al., 1999
	HL60 (50 μM) and TF1 (30 μM)	JAK/STAT	*In vitro*	HL60 / TF1	Induces cell cycle arrest	Ruela-de-Sousa et al., 2010
	40 μM, 20, 40 mg/kg	Akt, JNK	*In vitro, in vivo*	U937	Induces apoptosis	Budhraja et al., 2012
Melanoma	40 μM	caspase-3/ PARP/ ERK1/2 proteins/ p-AKT and p-mTOR	*In vitro*	A375, C8161	Inhibits proliferation and invasion; Induces apoptosis, and cell cycle arrest	Zhao et al., 2017a
	20 μM	FAK/ERK1/2	*In vitro*	A2058, A375	Inhibits metastasis	Hasnat et al., 2015
	20 μM, 150 mg/kg	MMP-2, MMP-9, VEGF, and Twist1	*In vitro, in vivo*	A375, G361	Inhibits metastasis	Cao et al., 2016
Prostate cancer	20 μM	cyclin D1, D2, and E; WAF1/p21	*In vitro*	LNCaP	Inhibits cell proliferation; Induces apoptosis	Gupta et al., 2002
	20 μM, 20,50 μg/kg	XIAP, c-IAP1, c-IAP2/ Bcl-xL and Bcl-2 and Bax protein	*In vitro, in vivo*	PC-3 and DU145	Induces cell cycle arrest and apoptosis	Shukla et al., 2014b
	20 μM	E-cadherin/ snail and vimentin	*In vitro*	DU145	Inhibited migration and invasion; cell cycle arrest	Zhu et al., 2015
	20 and 50 μg/kg	IKK – IκBα	*In vivo*		Inhibits tumorigenesis properties	Shukla et al., 2015b
	20 μM, 50 μg/ 50 μg/Kg	IKKα; NF-κB/p65	*In vitro, In vivo*	PC-3 and 22Rv1	Inhibits cell proliferation, invasion	Shukla et al., 2015a
	25 μM	Smad2/3 and Src/FAK/Akt	*In vitro*	PC3-M and LNCaP C4-2B	Inhibits cell proliferation and metastases	Mirzoeva et al., 2014
	25 μM	p21 and p27; caspases-8,−3 and TNF-α;	*In vitro*	PC3	Induces apoptosis and cell cycle arrest; suppresses stem cell migration	Erdogan et al., 2016
Lung cancer	20 μM	GLUT 1	*In vitro*	H1299 and H460	Inhibits cell proliferation; Induces apoptosis	Lee et al., 2016
	40 μM	PI3K/Akt	*In vitro*	A549	Inhibits cell proliferation, migration, invasion	Zhu et al., 2017
Breast cancer	40 μM	p-JAK1, p-JAK2 and p-STAT3; caspase-8, caspase-3; PARP	*In vitro*	BT-474	Inhibits cell proliferation; Induces apoptosis	Seo et al., 2015a
	40 μM, 5, 25 mg/kg	cyclin A, cyclin B, and CDK1;p21^WAF1/CIP1^;	*In vivo, In vitro*	MDA-MD-231	Induces cell cycle arrest	Tseng et al., 2017
	40 μM	caspase3, PARP and Bax/Bcl-2	*In vitro*	MDA-MB-231 and T47D	Inhibits cell proliferation; Indices apoptosis	Cao et al., 2013
	30 μM	IFN-γ-; PD-L1; STAT1	*In vitro*	MDA-MB-468 and 4T1	Enhances the immune responses	Coombs et al., 2016
	40 μM	p-JAK2 and p- STAT3; VEGF	*In vitro*	SKBR3	Induces apoptosis	Seo et al., 2015b
	60 μM	caspase-8, caspase-3 and PARP; JAK2 and STAT3	*In vitro*	MDA-MB-453	Inhibits cell proliferation; Induces apoptosis	Seo et al., 2014
Colorectal cancer	40 μM	Wnt/β-catenin	*In vitro*	SW480	Inhibits proliferation, invasion and migration	Xu et al., 2016
	25 μM	cyclin B1, Cdc2, and Cdc25c	*In vitro*	HCT116	Inhibits proliferation; Induces autophagy and apoptosis	Lee et al., 2014
	40 μM, 20 mg/kg	NEDD9	*In vitro, in vivo*	DLD1 and SW480	Inhibits proliferation, invasion and migration	Dai et al., 2016
	40 μM, 50 mg/kg	TAGLN; MMP-9; Akt	*In vitro, in vivo*	SW480, DLD-1, and LS174T	Inhibits proliferation, invasion and migration	Chunhua et al., 2013

### Apigenin in Induction of Cell Cycle Arrest

Another important and key feature of cancer is its uncontrolled and rapid cell division (Farhood et al., 2020). Therefore, targeting the growth of cancer cells is pivotal for suppressing cancer. One of the abnormalities in cancer cells is proliferation without paying attention to checkpoints of cell cycle. Notably, phytochemicals have demonstrated great potential in the activation of checkpoints and the induction of cell cycle arrest in cancer cells to limit their growth (Farooqi et al., 2019; Aggarwal et al., 2020). Based on the documentation presented, one of the prominent roles of apigenin is to modulate the cell cycle and block the cellular phase at the G2/M or G0/G1 checkpoint, which hinders cancer cell proliferation. In a study which was conducted to determine the effect of apigenin in human colorectal carcinoma HCT116 cells, it was shown that treatment with this flavone (0–50 μM) potentially inhibits cell growth through inducing cell arrest at the G2/M phase; it is associated with the suppression of the expression level of both cyclin B1 as well as both Cdc2 and Cdc25c which are cyclin B1 activating partners, and also an increase of the expression level of cell cycle inhibitors, p53 and p21^WAF1/CIP1^ (Lee et al., 2014). Also, in experiments performed by the Western blot technique, it was found that the expression levels of cyclin A, cyclin B, and cyclin-dependent kinase-1 (CDK1) were repressed by apigenin treatment in human breast cancer cell line MDA-MB-231. Based on the findings, apigenin (0–40 μM) led to the up-regulation of p21^WAF1/CIP1^ and enhanced the interaction of p21^WAF1/CIP1^ with a nuclear proliferating cell antigen (PCNA) preventing cell cycle development at the G2/M stage (Tseng et al., 2017).

The inhibitory effect of apigenin on the cell cycle can be related to its impact on genetic materials. Synthesis of genetic materials is a critical step for the proliferation of cancer cells, and any impairment or damage in DNA can lead to growth inhibition (Gourley et al., 2019). Apigenin follows a same method in suppressing the proliferation of cancer cells. It seems that in a time- and dose-dependent manner, apigenin (0–80 μM) causes DNA damage and encourages G2/M phase cell cycle arrest through ataxia telangiectasia mutated (ATM) modulation (Meng et al., 2017).

Cancer cells need high energy in order to grow and glucose transporter-1 (GLUT-1) participates in cancer proliferation by providing high energy *via* enhancing glucose uptake. Inhibition of GLUT-1 is a promising strategy in cancer therapy, and phytochemicals have demonstrated great potential in this way (Zambrano et al., 2019). Considering the functions of apigenin (10–160 μM) in adenoid cystic carcinoma (ACC), it causes G2/M-phase arrest, and ACC-2 cell growth and proliferation inhibition in a dose- and time-dependent manner *via* lessening the expression level of GLUT-1 (Fang et al., 2015).

As it was mentioned earlier, DNA damage is induced by apigenin to trigger cell cycle arrest. One of the ways to stimulate DNA damage is by providing oxidative stress. Enhancing the generation of reactive oxygen species (ROS) is associated with oxidative stress that subsequently, induces DNA damage in cancer cells, and inhibits their proliferation (Shuai et al., 2019; Srinivas et al., 2019). Through apigenin (12.5–100 μM) treatment in human papillary thyroid carcinoma BCPAP cells, G2/M cell cycle arrest occurred by down-regulating the Cdc25c expression level and also, the accumulation of ROS produced were stimulated which triggered DNA damage (Zhang et al., 2015).

Moreover, cell cycle arrest at the G0/G1 or S checkpoints can be induced by apigenin. Apigenin leads to G1 arrest of cell cycle progression in human prostate cancer LNCaP cells. The expression level of some proteins like cyclin D1, D2, and E and their activating partners CDK2, 4, and 6 were reduced noticeably, while the expression of p21^WAF1/CIP1^ and p27^KIP1^ were boosted simultaneously by apigenin treatment (10 μM). The induction of p21^WAF1/CIP1^ seems to be transcriptionally up-regulated and it was dependent on p53 (Gupta et al., 2002). In addition, cell cycle arrest at G0/G1 as well as G2/M checkpoints were triggered through treatment with apigenin (100 μM) in an oral squamous cell carcinoma cell line SCC-25. Also, it was related to the decreased expression of cyclin D1 and E, and the inactivation of CDK1 (Maggioni et al., 2013).

**Figure 2:**
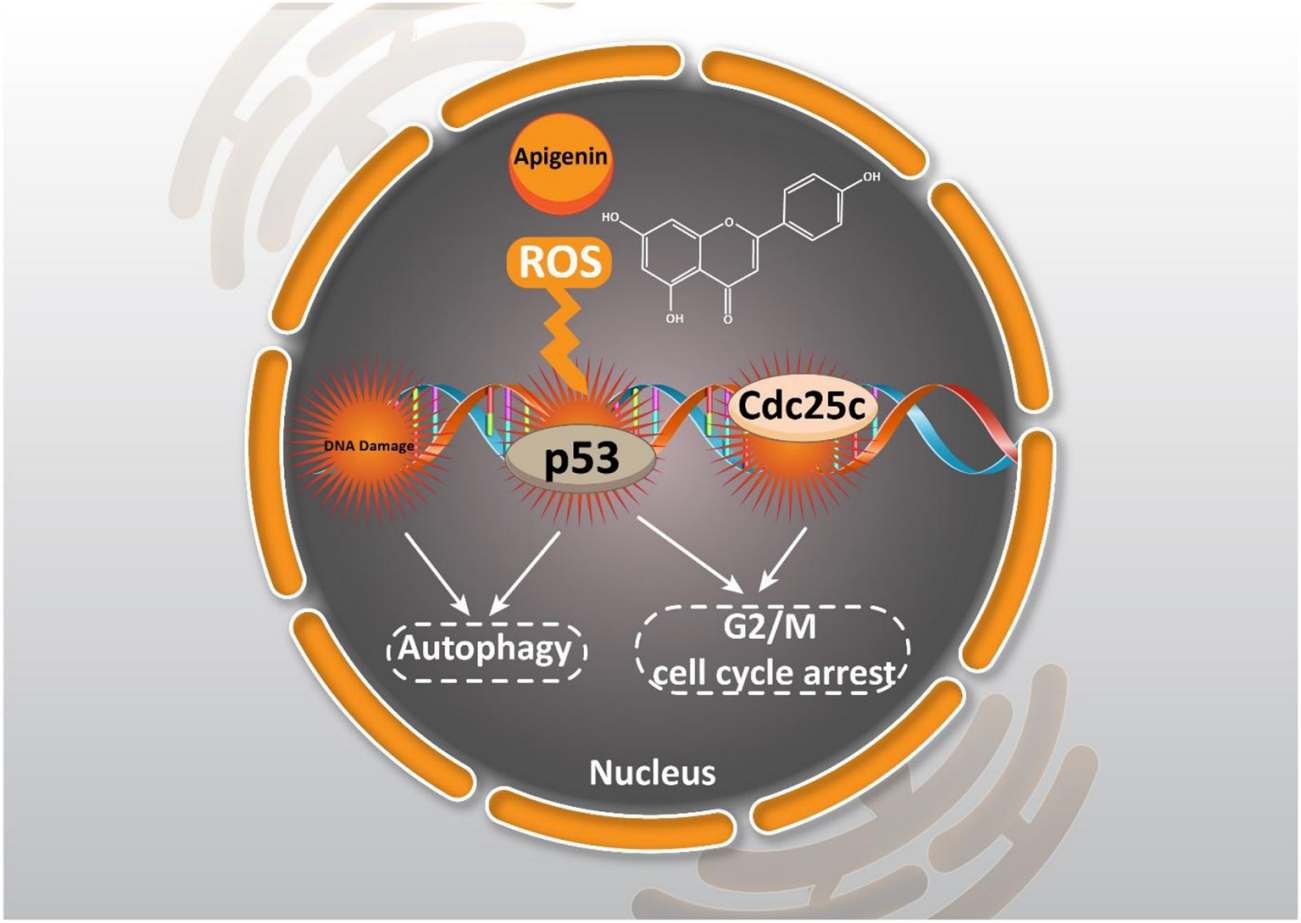
Effect of apigenin on autophagy in cancer. Apigenin affecting ROS generation, DNA damage, and cell cycle arrest could induce autophagy and cell cycle arrest.

Notably, cancer cells are able to acquire resistance to chemotherapy-mediated cell cycle arrest. Administration of apigenin is beneficial in suppressing chemoresistance, and sensitizing cancer cells in chemotherapy-mediated cell cycle arrest. It is held that apigenin (1–200 μM) is able to induce cell cycle arrest at the G2/M phase. Notably, in this study, it was demonstrated that apigenin induces cell cycle arrest in a dose-dependent manner, so that the highest concentration of apigenin (200 μM) induces cell cycle arrest at the S phase and the highest inhibitory effect on the proliferation of imatinib-resistant cancer cells (Solmaz et al., 2014). Taking everything into account, the results presented reveal that apigenin may regulate cell cycle progression in a dose-dependent and/or cell line specific manner (Figure 2).

### Apigenin and Programmed Cell Death

Apoptosis, a type of programmed cell death, involves energy-dependent cascade events and diverse distinct morphological characteristics (Elmore, 2007; Mortezaee et al., 2019d). There are two main cascades involved in the apoptosis process: the extrinsic (death receptor) pathway as well as the intrinsic (mitochondrial) pathway (Chong et al., 2020). Apoptosis is a vital procedure in which undesirable cells are removed under physiological circumstances (Sun et al., 2020). An important feature of cancer cells that distinguishes them from normal cells is their escape from apoptosis (Deng et al., 2020). Therefore, one of the imperative strategies to fight cancer cells and to treat cancer is to stimulate apoptosis of these cells, in which they target apoptotic pathways with chemotherapeutic agents (Liu et al., 2020; Maruszewska and Tarasiuk, 2020). It was revealed that apigenin can be considered as an influential factor in inducing apoptosis through the intrinsic or extrinsic pathway of human cancer cells.

**Figure 3:**
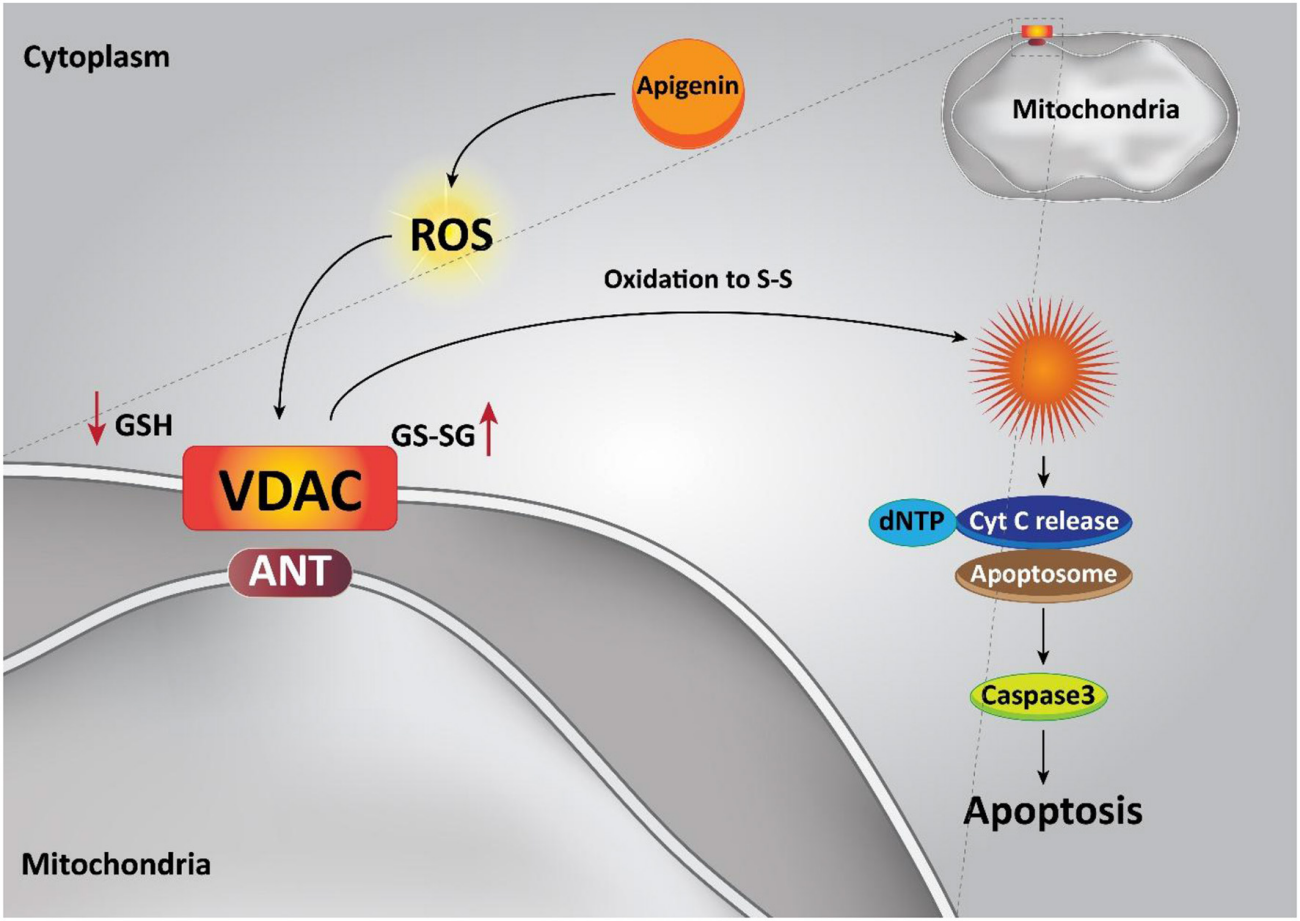
Effect of apigenin on the intrinsic pathway of apoptosis in cancer cells.

The intrinsic apoptotic pathway is regulated via the Bcl-2 family of proteins, such as Bcl-2, Bcl-xL, Bcl-w, and Mcl-1, which suppress this pathway, while Bad, Bak, Bax, Bid, and Bim cause apoptosis (Billard, 2012; Vela and Marzo, 2015; Zheng et al., 2016). Pro-apoptotic protein up-regulation and/or pro-survival members down-regulation are the functions of apigenin (20 μM), in that way the intrinsic apoptotic cascade is induced. An apoptosis event was caused through the treatment of the androgen-refractory human prostate cancer cell lines PC-3 and DU145 and also, it led to a decrease in cell feasibility triggered by a decline in Bcl-2 and Bcl-xL and an enhancement in the active form of the Bax protein, attended by dose-dependent prevention of XIAP, c-IAP1, c-IAP2, and survivin proteins (Shukla et al., 2014b). In addition, apigenin treatment (0–100 μM) in human promyelocytic leukemia HL-60 cells leads to a diminution in mitochondrial outer membrane potential, releasing cytochrome c from the mitochondria into the cytosol, and encouraging both procaspase-9 processing and cell apoptosis through the intrinsic apoptotic pathway (Wang et al., 1999). Furthermore, apigenin has also been reported to induce apoptosis by altering the ratio of pro-apoptotic to pro-survival mitochondrial proteins. Ratio of Bax / Bcl-2 in favor of cell apoptosis is improved in prostate cancer cells by the means of apigenin (10 μM) (Gupta et al., 2002). Obviously, apigenin alone is capable of inducing mitochondrial-dependent apoptosis in various kinds of cancer cells (Figure 3) (Das et al., 2012; Lim et al., 2016; Wang and Zhao, 2017).

**Figure 4:**
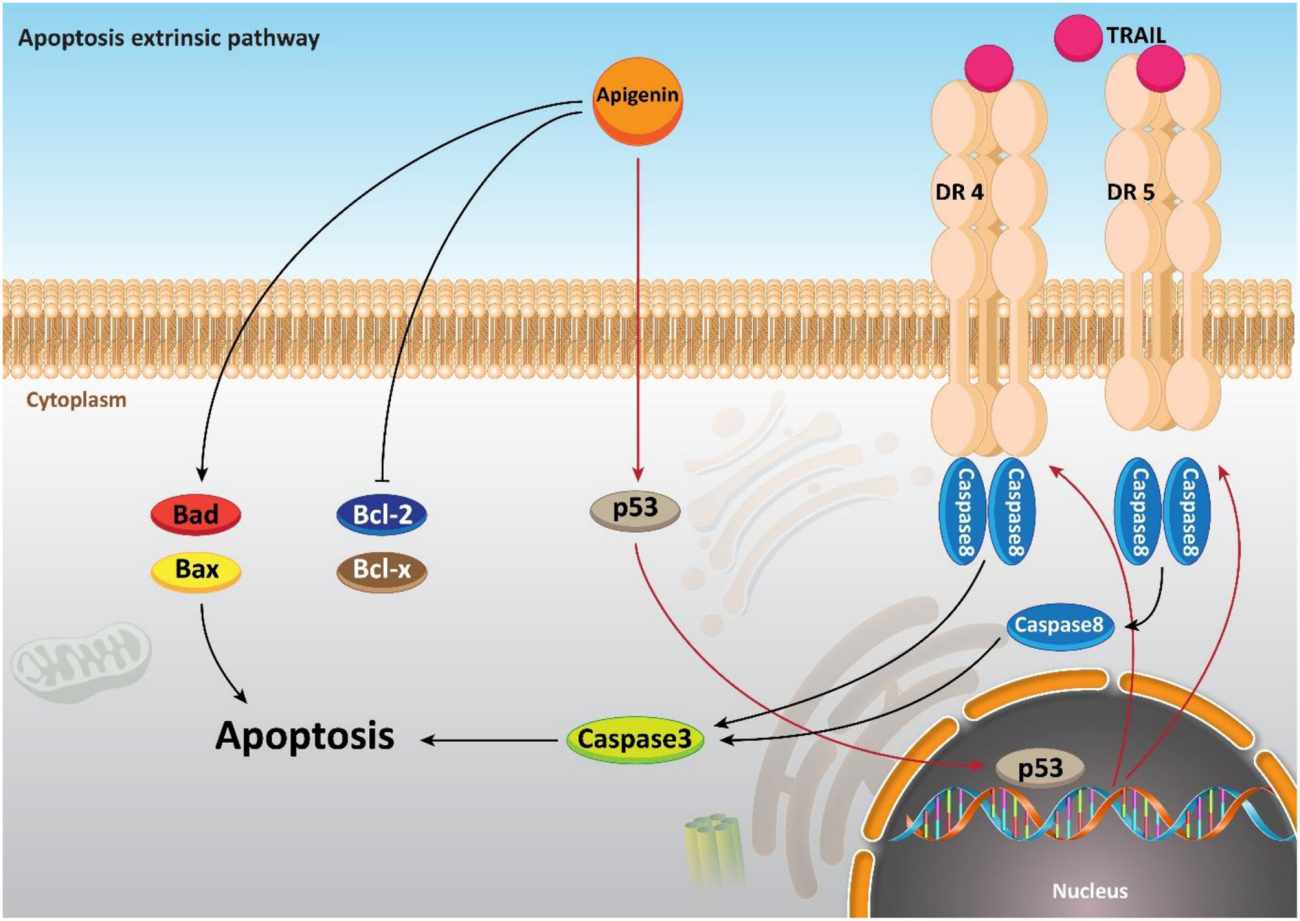
The effect of apigenin on extrinsic pathway of apoptosis.

Moreover, it seems that apigenin is advantageous in boosting chemotherapy-mediated cell apoptosis *via* affecting mitochondrial proteins. It is said that the administration of apigenin (20 μM) enhances the expression of the pro-apoptotic factor Bim, while it decreases expression of Mcl-1. So, co-administration of apigenin with a Bcl-2 inhibitor Navitoclax promotes mitochondria-mediated cell apoptosis (Shao et al., 2013). In addition to its role in inducing an intrinsic apoptotic pathway, apigenin has a function in inducing the apoptosis process in cells through an extrinsic apoptotic pathway or even both the extrinsic and intrinsic pathways. To further examine the role of apigenin in human breast cancer BT-474 cells, Seo et al. carried out a series of experiments and it was reported that apigenin treatment neither affected the levels of Bcl-2 and Bax nor declined the mitochondrial membrane potential. On the other hand, extrinsic, caspase-dependent apoptosis created by up-regulating the levels of cleaved caspase-8 and cleaved caspase-3 are induced through this compound treatment (20, 40, and 80 μM) (Seo et al., 2015a). Chen et al., studied the effects of apigenin (0–160 μM) on non-small cell lung cancer (NSCLC) cells and pointed out that in a p53-dependent manner, the levels of death receptor 4 (DR4) and death receptor 5 (DR5) were up-regulated. Thus, sensitizing NSCLC cells to TRAIL-induced apoptosis. Furthermore, it was revealed that exposing lung cancer cells to apigenin (0–160 μM) induces apoptosis *via* the up-regulation of pro-apoptotic factors Bad and Bax, and the down-regulation of anti-apoptotic factors Bcl-xl and Bcl-2 (Chen et al., 2016). In addition, a good example of the role of apigenin in both intrinsic and extrinsic apoptosis pathways is observed in human keratinocytes and organotypic keratinocytes, which increases UVB-induced apoptosis through both pathways. Bax localization and cytochrome c release were altered by apigenin (0, 10, and 20 μmol/L). Overexpression of the pro-survival protein Bcl-2 and the dominant-negative form of Fas-associated death domain protected against apigenin-induced apoptosis (Figure 4) (Abu-Yousif et al., 2008).

Autophagy is a process known as type 2 non-apoptotic cell death (Hazari et al., 2020). The sequestration of cytoplasmic material into vacuoles for bulk degradation by lysosomal enzymes is the feature of this regulated mechanism (Galluzzi and Green, 2019). In other words, a cell digests its own cytoplasmic materials within lysosomes and results in the decomposition of macromolecules through this conserved dynamic process (Korolchuk and Rubinsztein, 2011; Chaabane et al., 2013; Yang and Klionsky, 2020). To date, there is a growing body of evidence to suggest that the association between autophagy and cancer is complicated and contradictory (Wen and Klionsky, 2019; Galluzzi and Kroemer, 2020). Autophagy performs various functions in the body; during starvation, this mechanism serves as a cell survival pathway by preparing recycled metabolic substrates as well as keeping energy homeostasis (Sameiyan et al., 2019). Besides, it results in cell death, in association with an apoptosis pathway or as a backup mechanism (Nazim et al., 2020). Apigenin-induced autophagy was first detected in erythroleukemia TF1 cells. Apigenin exposure (0–200 μM) leads to the onset of autophagy lacking apoptosis (Ruela-de-Sousa et al., 2010). Since then, more evidence demonstrated that autophagy could be triggered by apigenin and also under diverse conditions, it acts as tumor suppressive or tumor protective (Sung et al., 2016; Salmani et al., 2017).

Investigating the impact of apigenin (20 μM) on human keratinocytes, Tong et al. reported that autophagy was induced through AMPK activation by this chemo-preventive bioflavonoid (Tong et al., 2012). Cao et al. performed a similar series of experiments to show that in human breast cancer T47D and MDA-MB-231 cells treated with apigenin (0–80 μM), both apoptosis and autophagy pathways were triggered through the accumulation of acidic vesicular organelles (AVOs) and LC3-II, a marker of Atg5/Atg7 dependent autophagy. In addition, further studies have revealed that apigenin-induced apoptosis is significantly enhanced during treatment with the 3-methyladenine (MA) autophagy inhibitor. It shows that autophagy triggered by apigenin performs a tumor protective role in apigenin-caused cytotoxicity (Cao et al., 2013). Similarly, Lee et al. demonstrated that in human colon cancer HCT116 cells, apigenin (0–50 μM) simultaneously induces both apoptosis as well as autophagy. Autophagy played a cell protective role in apigenin-induced cell apoptosis as well (Lee et al., 2014).

Beclin-1 is able to regulate the dynamic autophagy procedure through the formation of autophagosomes (Liang et al., 2019; Vega-Rubín-de-Celis, 2019). In various kinds of cancers including solid Ehrlich carcinoma, Beclin-1 is regularly down-regulated. Gaballah et al. published a paper in which they described that combining 5-FU with apigenin (100 mg/kg/day) pointedly improved Beclin-1 compared to the vehicle-treated control mice (Gaballah et al., 2017). Furthermore, according to the study of Wang et al. autophagy is induced in macrophages during apigenin treatment (10, 25, and 50 μM), as evidenced by the further regulation of Beclin-1, Atg5, Atg7, and the presence of LC3-II. Further, based on experiments, inhibition of autophagy by 3-MA pretreatment remarkably boosted apigenin-induced apoptosis. Also, signifying that the autophagy caused by apigenin protected macrophages from apigenin-induced cytotoxicity (Wang et al., 2015).

In contrast, through investigations into human papillary thyroid carcinoma BCPAP cells, it was discovered that treatment with apigenin (12.5, 25, and 50 μM) leads to autophagic cell death following p62 degradation, Beclin-1 accretion as well as LC3 protein conversion. Interestingly, additional examination demonstrated that apigenin-induced cytotoxicity was significantly protected *via* co-treatment with 3-MA, which indicated that apigenin-induced autophagy here is more likely to be a tumor suppressor (Zhang et al., 2015). Together, according to cancer cell types, autophagy has a diverse role in apigenin-induced cytotoxicity.

In most reports, the function of apigenin-triggered autophagy is to mediate the acquired resistance of cancer cells versus cell apoptosis, evidenced as improved cell apoptosis encouraged by apigenin when in cotreatment with autophagy inhibitors. Under this circumstance, the autophagy performs cytoprotective tasks in apigenin-induced cytotoxicity in cancer cells. In contrast, in human papillary thyroid carcinoma BCPAP cells, autophagy operates as an executioner through encouraging autophagic cell death (Zhang et al., 2015).

As it was mentioned, apigenin is capable of inducing both autophagy and apoptosis, as major arms of programmed cell death (PCD). However, apigenin-mediated autophagy results in the enhanced survival of cancer cells, and their resistance into chemotherapy. This is due to the dual role of autophagy in cancer cells. Increasing evidence demonstrates that autophagy works like a double-edged sword in cancer cells, and it may function as a pro-survival or pro-death mechanism (Huang et al., 2019b; Wang et al., 2019b). In the case of apigenin, autophagy induction is correlated with an increase in the survival of cancer cells, and its down-regulation by autophagy inhibitors can pave the road to effective cancer therapy.

### Apigenin and Cancer Metastasis

Tumors are divided into benign and malignant types based on a number of criteria including location and growth characteristics, and tissue origin. Lack of migration is a prominent feature of benign tumor cells. Unlike malignant tumor cells which are highly unstable and capable of metastasizing and attacking other tissues to cause more lesions, benign tumor cells grow only at the primary site of the tumor and cause lesions and can be eliminated through clinical surgery. Most patients die with varying grades of tumor metastasis, not in the clinical practice of the primary disease. Currently, metastases, accompanied by chemoresistance development as well as tumor recurrence, are still key obstructions in operative cancer treatment (Murugan, 2019; Zhang, 2019; Zhuang et al., 2019). It was explored that for *in vitro* cancer cells and *in vivo* animal models, apigenin can suppress cancer cell migration and invasion (Chien et al., 2019; Lee et al., 2019a; Tong et al., 2019).

Plant derived-natural compounds are able to target molecular signaling pathways involved in cancer growth and metastasis (Zhang et al., 2019; Liao et al., 2020). Accumulating data exhibit that the PI3K/Akt signaling pathway plays a significant role in cancer growth and metastasis. Inhibition of the PI3K/Akt signaling pathway inhibits the malignant behavior of cancer cells, and restricts their migration (Huang et al., 2019a; Zheng et al., 2019). Tumor cell invasion and migration in a dose-dependent manner are repressed via apigenin (0-20 μM) in prostate cancer DU145 cells (Zhu et al., 2015). Concerning the effect of apigenin on A375 and C8161 melanoma cell lines, it was found that 40 μM of this compound remarkably prevented cell migration and invasion through impacting the AKT/mTOR pathway (Zhao et al., 2017a). Also, an experiment of apigenin treatment (0–40 μM) in a human A549 lung cancer cell line revealed that this compound was able to arrest Akt phosphorylation and target the PI3K/AKT signaling pathway, leading to anti-migration and anti-invasion effects (Zhou et al., 2017). In a study conducted by Dai et al., it was shown that apigenin (0–50 μM) could inhibit cell migration, invasion, and metastasis through regulating the NEDD9/Src/AKT cascade in the colorectal cancer cell lines DLD1 and SW480 (Dai et al., 2016).

**Figure 5:**
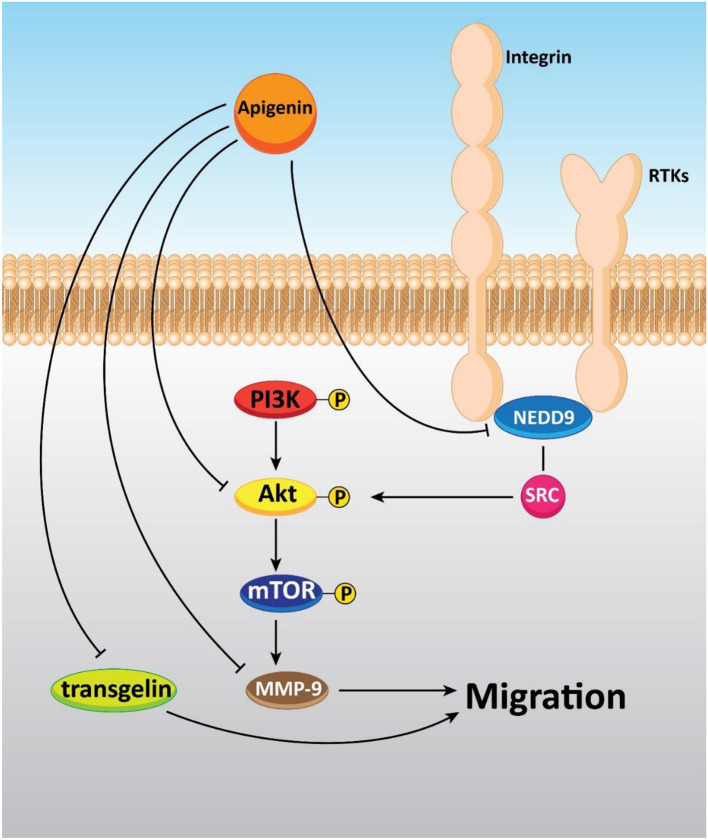
The inhibitory effect of apigenin on the metastasis of cancer cells.

Moreover, *in vitro* study has shown that apigenin lowered the migration and invasion of cancer cells by decreasing FAK expression in human ovarian cancer A2780 cells. Also, further experiments have revealed the spontaneous metastasis suppression of A2780 cells implanted into the ovary of nude mice *in vivo* by apigenin treatment (0–40 μM) (Hu et al., 2008). Additionally, cell proliferation and migration were prohibited through apigenin exposure by up-regulating and down-regulating transgelin and MMP-9 expression, respectively via decreasing the phosphorylation of Akt. Therefore, tumor growth and metastasis to the liver and lung were repressed by apigenin treatment (Figure 5) (Lieben, 2017).

## Apigenin-Loaded Nanovehicles

Overcoming poor bioavailability and low solubility of apigenin requires using carriers for targeted delivery of apigenin that remarkably enhances the anti-tumor activity of apigenin (Mahmoudi et al., 2019). Fortunately, different nanocarriers were designed for the delivery of apigenin and for reducing the survival and viability of cancer cells. On the other hand, as mentioned before, resistance of cancer cells to chemotherapy is an increasing challenge in the field of cancer therapy. Hence, using carriers for protecting and delivering of biotherapeutic agents not only ameliorates its anti-tumor activity but also sensitizes cancer cells to the inhibitory effects of chemotherapeutic agents (Mahmoudi et al., 2019; Jamaledin et al., 2020).

**Figure 6:**
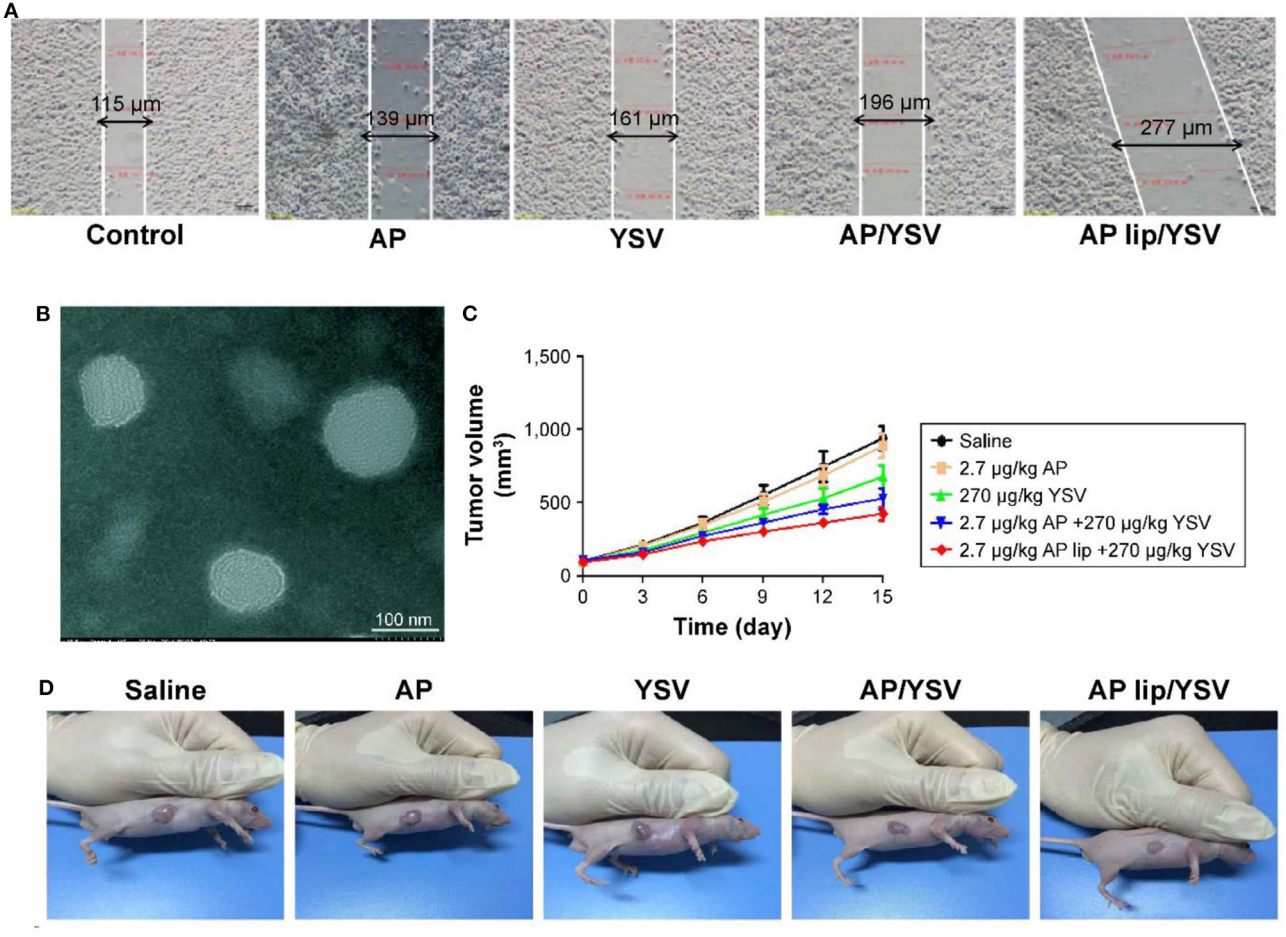
**(A)** Synergistic effects of apigenin-loaded TPGS liposomes and tyroservatide (YSV) in A549 cells. **(B)** Transmission electron microscopy (TEM) image of apigenin-loaded D-alpha-tocopheryl polyethylene glycol (TPGS) liposomes. Diagram of tumor volumes **(C)** and morphology **(D)** after 15 days. Reprinted with permission from Jin et al. (2017).

In light of this, several sorts of nanosized vehicles were exploited to encapsulate and liberate apigenin at the targeted site. Liposomes are ideal candidates for delivery of anti-tumor drugs with satisfactory results at preclinical experiments. Since liposomes contain a hydrophilic core and a hydrophobic bilayer, this nanocarrier can be applied for the delivery of both hydrophobic and hydrophilic drugs (Mickova et al., 2012; Sen and Mandal, 2013). In respect to the capability of liposomes in encapsulating several drugs, they can significantly improve the therapeutic effects of their cargo (Hu and Zhang, 2012; Gowda et al., 2013; Sen et al., 2014). It is noteworthy that liposomes were used for the co-delivery of apigenin and 5-fluorouracil in colorectal cancer therapy. Using liposomes is correlated with an increase in the cytotoxicity of apigenin and 5-fluorouracil against colorectal cancer cells (Sen et al., 2019). Apigenin- and 5-fluorouracil-loaded liposomes are able to efficiently inhibit angiogenesis and induce apoptosis in colorectal cancer cells, leading to a decrease in the proliferation and viability of colorectal cancer cells. It is worth mentioning that using liposomes for the delivery of apigenin and 5-fluorouracil significantly promotes the capability of these anti-tumor agents in the up-regulation of AMPK, and the inhibition of the Warbrug effect (Sen et al., 2019). The study demonstrates that such nanocarriers enhance the anti-tumor activity of apigenin and the efficacy of chemotherapy; in which, it exerts a more inhibitory effect on the proliferation of cancer cells by the induction of apoptosis, and by disrupting glycolysis metabolism, and promoting the efficacy of targeting molecular pathways. Apigenin-loaded liposomes have demonstrated great potential in disrupting the cell membrane of cancer cells which is an underlying mechanism for stimulation of cell cycle arrest at the G2/M phase (Banerjee et al., 2017). In addition to colorectal cancer, apigenin/tyroservatide-loaded liposomes were applied for lung cancer therapy. It was shown that these delivery systems are able to remarkably stimulate apoptosis and cell cycle arrest at the G2 phase in A549 cells (Figure 6) (Jin et al., 2017). This is due to providing targeted delivery and enhancing the accumulation of apigenin in cancer cells.

Apigenin-loaded nanoparticles were applied in the treatment of hepatocellular carcinoma that resulted in enhanced cytotoxicity of apigenin against cancer cells. This is due to increased availability of apigenin in blood circulation and accumulation in the liver (Bhattacharya et al., 2018). Interestingly, nanocarriers enhanced cytotoxicity of apigenin against cancer cells without affecting normal cells (high biocompatibility) (Jangdey et al., 2019).

**Figure 7:**
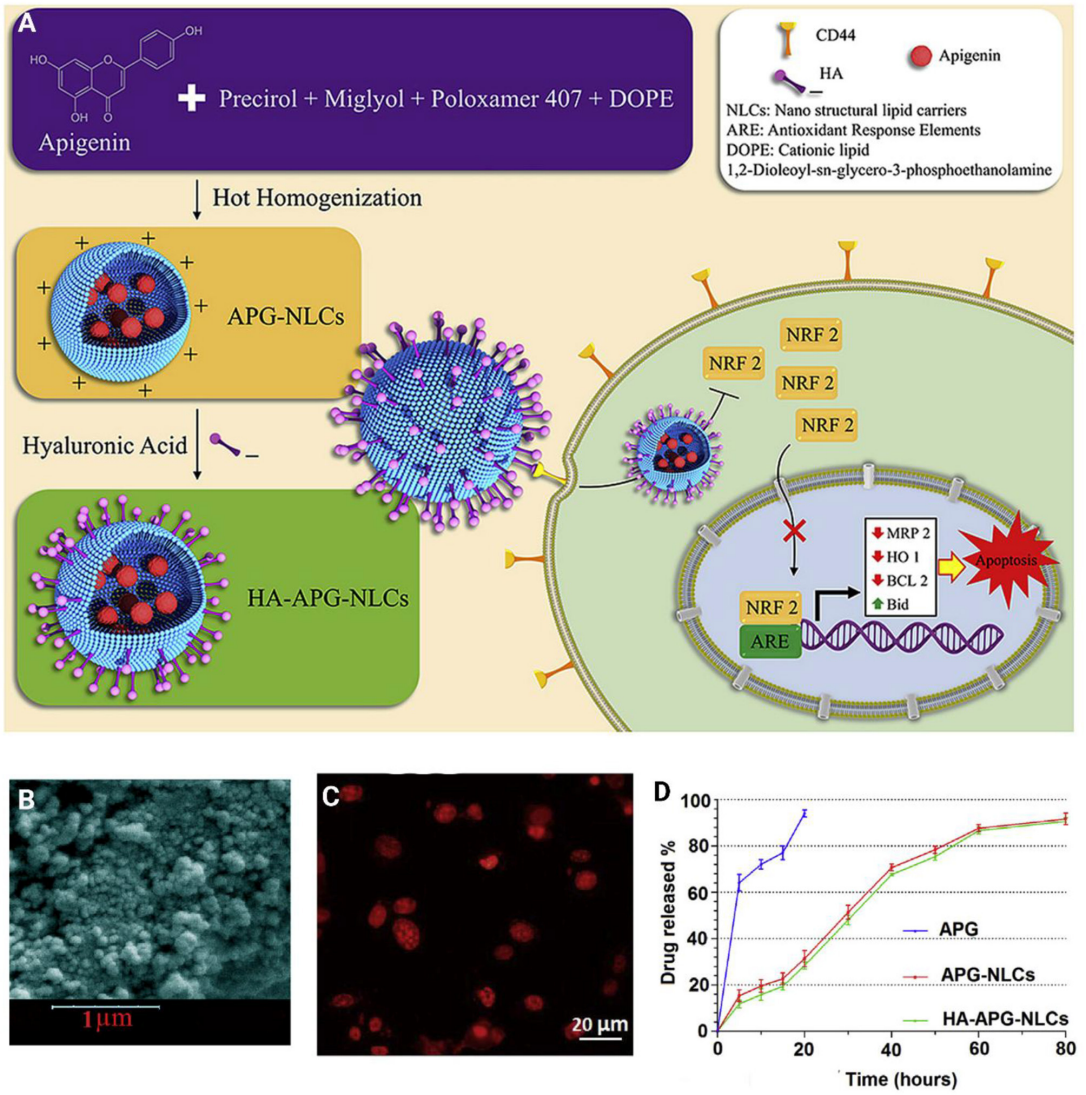
**(A)** Schematic illustration of hyaluronic acid-functionalized nanostructured lipid carriers (HA-NLCs) containing apigenin. **(B)** SEM images HA-NLCs. **(C)** *In vitro* internalization of Rhodamine B encapsulated apigenin-nanostructured lipid carriers. **(D)** *In vitro* drug release of apigenin solution (APG), APG encapsulated-NLCs, and HA-NLCs. Reprinted with permission from Mahmoudi et al. (2019).

Surface functionalization of nanoparticles significantly enhances their capability to target cancer cells. Surface modification can be directed toward targeting special receptors undergoing overexpression in cancer cells. For instance, a cluster of the differentiation protein CD44 shows high expression in cancer cells, and sodium hyaluronate can be used for surface modification of nanoparticles, and targeting CD44 in cancer cells (Dosio et al., 2016). Such a strategy was employed for the delivery of apigenin. It was reported that sodium hyaluronate nano-assemblies are capable of enhancing the accumulation of apigenin in lung cancer cells *via* targeting CD44 and providing receptor-mediated endocytosis (Zhao et al., 2017b). The surface decoration of nanocarriers using hyaluronic acid (HA) offers the possibility of targeting CD44 receptors. On the other hand, HA functionalization of nanovehicles can indeed change physicochemical properties, alter stability, toxicity, and influence nanoparticle biodistribution and efficiency *in vitro* and *in vivo* (Wang et al., 2016; Xu et al., 2017; Alves et al., 2019). In light of this, HA decorated-lipid nanoparticles were employed for the delivery of anti-tumor therapeutics (Figure 7) (Mahmoudi et al., 2019). The finding showed that co-delivery of apigenin and docetaxel impose synergistic anti-cancer effects toward A549 cells. In addition, surface coating lipid nanocarriers with hyaluronic acid enhanced the cellular uptake in comparison with pristine lipid NPs (Mahmoudi et al., 2019). Other carrier systems based on HA (e.g., chemically crosslinked hydrogel nanocomposites or *in situ* gel at body temperatures, known as thermosensitive hydrogel) are other valuable options to deliver biotherapeutics to the human body (Kim et al., 2018; Makvandi et al., 2019, 2020; Tan et al., 2019).

Taking these findings into account, nanocarriers are promising candidates in the delivery of apigenin for cancer therapy. They are capable of exponentially improving the cytotoxicity of apigenin toward tumor cells. Besides, they promote bioavailability and cellular uptake of apigenin, leading to an increase in the therapeutic efficacy of apigenin (Das et al., 2013; Jangdey et al., 2017).

## Apigenin and Pancreatic Cancer Therapy

Dietary cancer prevention and chemoprevention are two main diet-related prevention strategies in cancer studies (Singletary, 2000). Cancer chemoprevention uses synthetic, natural, or biologic chemicals in the pharmacologic intervention struggling against carcinogenesis (Tsao et al., 2004). However, dietary cancer prevention is defined as a modified pattern of food consumption along with lifestyle alteration that reduces cancer risk (Schatzkin and Kelloff, 1995; Singletary, 2000). Recently, food-based strategies for pancreatic cancer prevention have been studied using epidemiological evidence. These studies demonstrated an inverse relationship between vegetable and fruit consumption and the risk of pancreatic cancer development (Larsson et al., 2006; Polesel et al., 2010).

Apigenin is a flavone usually found in vegetables and fruit such as citrus (e.g., grapefruit) (Cirmi et al., 2016). The fruits and vegetables enriched in apigenin have shown an anti-cancer effect. The outcomes are primarily attributed to their anti-inflammatory and antioxidants effects (Madunić, Madunić et al., 2018). Apigenin has free radical scavenging and anti-inflammatory characteristics. It is also considered as an anti-cancer agent, reducing the proliferation of cancer cells, while having no effects on normal cells (Cirmi et al., 2016). Apigenin has shown growth inhibitory properties in breast cancer *via* apoptosis promotion *via* (a) activation of the caspase cascade; (b) blocking NF-κB and STAT3 signaling in breast cancer cells with HER2-overexpression; (c) eliminating both the PI3K and Akt kinase activity and modulation of the p14ARF-Mdm2-p53 pathway (Way et al., 2004; Agrawal et al., 2006; Choi and Kim, 2009; Seo et al., 2012). We have summarized anti-pancreatic cancer effects of apigenin and its glycosides below.

### Molecular Pathways and Mechanisms

Apigenin, due to the stimulation of the production of reactive oxygen species (ROS) (Shukla and Gupta, 2008) in solid and hematological cancers, has shown some anti-cancer properties (Granato et al., 2017). By way of illustration, one of the most common strategies which is applied in both conventional and non-conventional radio- and chemo-therapies is stimulating the ROS generation with the aim of killing the cancer cells (Yang et al., 2018). Several factors are involved in balancing the level of the ROS in cells. NADPH-oxidases and mitochondrial complexes which produce ROS, enzymes which have intervener roles in antioxidant responses, e.g., superoxide dismutase (SOD) and catalase, and enzymes involved in detoxifying of ROS such as glutathione S-transferase (GST) are some well-known examples of these factors (Kumari et al., 2018). The regulation in the expression level of the anti-oxidant enzymes is mostly controlled by the transcription nuclear factor erythroid 2 like2 (NRF2) (Chatterjee et al., 2013). Therefore, redox resetting for surviving tumor cells in anticancer drug treatment conditions is usually induced by the tumor cells themselves (Liu et al., 2016). NRF2-induced pathways have important roles in the chemotherapeutic resistance of cancer cells (Rojo de la Vega et al., 2018).

Tumor suppressor P53 is a prominent factor in responses to anti-tumor therapies. The functional p53 pathway prevents tumor development and growth; therefore, the p53 gene is mutated in most of the tumors (Mantovani et al., 2019). The “gain of function” mutation which prompt tumor cells to grow, metastasize, and resist therapies is one of the most common mutations in P53 genes (Bellazzo et al., 2018). Tp53 mutations (mutp53) are very common in PDAC (Muller and Vousden, 2014). Mutp53 proteins usually would not become degraded due to obtaining a mis-folded conformation; therefore, hyper-stable proteins may accumulate in tumors (Alexandrova et al., 2015). The most well-known factors affording mutp53 stability are cellular chaperone heat shock proteins (HSP70 and HSP90) which bind to mutp53 and prevent the degradation of this protein with a double minute 2 homolog (MDM2) protein. The HSP90 expression level is increased in cancer cells in order to assist in cancer cell survival; hence, HSP90 may be a potential target in cancer therapy (Solarova et al., 2015). The heat shock factor 1 (HSF1) is the most predominant factor which controls the heat shock response. HSF1 by contacting mutp53 may enhance the HSF1-induced transcription program, which up-regulates heat shock proteins (HSPs) in a positive feed-forward loop that results in more maintenance of mutp53 (Li et al., 2014). There may be an interaction between NRF2 and HSF1 in which HSP and P62 are their mutual targets (Dayalan Naidu et al., 2015). Furthermore, mutp53 may cooperate with the NRF2 with the aim of moderating the NRF2-mediated anti-oxidant response (Lisek et al., 2018). It was shown that NRF2 induced pancreatic carcinogenesis in mice with k-ras mutations and mutp53 (Hamada et al., 2017).

Above all mutp53 is an essential target for anticancer therapy. Accordingly, several mutp53 approaches were examined in recent years such as applying strategies to mutp53 degradation and/or repetition of the wild-type p53 (mutp53 as a dominant negative effect prevents wild-type p53) (Schulz-Heddergott and Moll, 2018). One possible strategy is to inhibit mutp53 by autophagy (Garufi et al., 2014, 2015). Another one is to use the zinc effect as a modifier in the mutp53 protein conformation, or through natural compound capsaicin (Garufi et al., 2016). Mutp53 despite the wtp53 suppresses autophagy which in turn inhibits mutp53 degradation (Cordani et al., 2016). Realizing the interaction between mutp53 and autophagy is essential for an effective anticancer therapy. The effect of apigenin in the cytotoxicity of PaCa44 and Panc1 cancer cells with various p53 mutations was assessed (Moore et al., 2001). As confirmed by an experiment on the apigenin effect on the toxicity of two pancreatic cell lines, PaCa44 and Panc1, which harbor various p53 mutations, by Montani et al. (Gilardini Montani et al., 2019). It was shown that the cytotoxic effect of apigenin on the Panc1 cell line is higher in comparison to its effect on PaCa44 cell lines. It was shown that the stronger cytotoxic effect of apigenin (6, 12.5, 25, and 50 μM) is due to a decrease in HSP90 and mutp53 expression, higher amounts of intracellular ROS, and mTORC1 suppression. In this study, it was recommend that targeting mTOR-mutp53-NRF2-p62-HSP90 molecules may be useful for overcoming the chemo-resistance of PC to apigenin (Gilardini Montani et al., 2019).

It was shown that up-regulation of CK2 results in the development of lymphomas and leukemia (Landesman-Bollag et al., 1998; Channavajhala and Seldin, 2002; Seldin et al., 2008). CK2-dependent signaling pathways are inhibited by apigenin. Several biological activities were attributed to apigenin such as anti-oxidant, anti-carcinogenic, anti-proliferative, and anti-inflammatory activities (Patel et al., 2007). Recently, several experiments focused on applying apigenin as a chemo-preventive agent in various cancers (Mafuvadze et al., 2012). Apigenin may lead to changes in regulatory T cells and effector T cells growth of murine PC (Nelson et al., 2015).

From a dysregulation which was shown between phosphatase 1 (PP1) and Casein Kinase II (CK2), it was suggested that higher CK2 expression controls the stability of the Ikaros. Furthermore, it was shown that down-regulation of the Ikaros leads to a reduction in CD4^+^ and CD8^+^ T cell percentages but augmented CD4^+^CD25^+^ Tregs in tumor-bearing (TB) mice (Nelson et al., 2017).

In a study, naïve mice splenocytes were treated with apigenin *in vitro* when Panc02 cells were present which lead to an evaluation of the Ikaros expression, like the activity of the proteasome inhibitor MG132. TB mice cells were treated with apigenin. *In vivo* results showed a decrease in tumor size and an inhibition of the splenomegaly. Furthermore, apigenin (10 and 20 μM) treatment leads to the reestablishment in production of a few Ikaros isoforms, which are probably responsible for the mild prevention of the CDK2 function in splenocytes of the TB-apigenin mice. Complementary to the incomplete reestablishment of the Ikaros expression, the percentage of the CD4+ and CD8+ T cells have shown a considerable growth and the percentage of Tregs have fallen into a significant decline in TB-apigenin mice. Moreover, CD8+ T cells from TB-apigenin mice compared to TB mice have shown additional production of the IFN-γ and splenocytes of the TB-apigenin mice are more susceptible to allogeneic CD8+ T cell responses. These findings offer more support for the idea that Ikaros in a pancreatic cancer model is controlled by CK2, and these results have shown that apigenin is a potential therapy for murine PC (Nelson et al., 2017).

Recently it was shown that hypoxia-inducible factor-1 α (HIF-1α) is targeted by apigenin in several cancers such as, ovarian cancer, prostate cancer, and lung cancer (Osada et al., 2004; Liu et al., 2005). HIF-1α is a prominent transcription factor for the transcription of the genes which are responsible for tumor development and invasion under hypoxic conditions (Melillo, 2006). An increase in the expression level of HIF-1α is revealed in several cancers which is a contributory factor for drug resistance and higher mortality (Birner et al., 2001; Koukourakis et al., 2006).

Hypoxia stimulates some changes in cellular metabolism, resistance to apoptosis, and induces angiogenesis in PC cells lines (Garcea et al., 2006). Pancreatic carcinomas compared to benign tumors show higher levels of the expression of the HIF-1α protein, more tumor proliferation, and less tumor differentiation (Mabjeesh and Amir, 2007).

The HIF is a protein consisting of two αβ heterodimers. The HIF-1α expression is stimulated by hypoxic conditions. The HIF-1β is constantly expressed (Garcea et al., 2006). Interaction of the HIF-1 αβ heterodimer with the hypoxia response element (HERs) leads to the stimulation of transcriptional activity. In physiological conditions, HIF prolyl hydroxylases hydroxylated the two prolyl residues of the HIF-1α protein and subsequently interaction between this protein and the von Hippel-Lindau (VHL) E3 ubiquitin ligase complex leads to proteasomal destruction of HIF-1α (Ivan et al., 2001). On the other hand, under hypoxic conditions, as the HIF prolyl hydroxylases are inactivated, the HIF-1α, which is accumulated, dimerized with HIF-1β which leads to hypoxic response gene transcription (Garcea et al., 2006).

As a result of the increase in the expression level of the HIF-1α gene in pancreatic cancer, several down-stream genes are subsequently transcriptionally activated. Production of these down-stream genes are necessary for angiogenesis, for example, the vascular endothelial growth factor (VEGF) and also, for glycolysis, the GLUT-1 glucose transporter (Lin et al., 2016; Zambrano et al., 2019).

VEGF as a secreted protein is an inducer for tumor vessel growth. The poor survival of patients with PC, low survival after the surgery, and subsequently hepatic metastasis are directly proportional to the serum level of the VEGF (Karayiannakis et al., 2003). PC cells which were treated by VEGF show more development and knock-out of the VEGF in pancreatic tumors of animal models leads to a decrease in vascularity and growth (Inoue et al., 2002).

The up-regulation of the glucose transporter (GLUT-1) is directly proportional to the poor prognosis in several cancers including ovarian, gastric, breast, and colorectal carcinomas (Zambrano et al., 2019). Gene expression of the GLUT-1 is correlated to the cancer metastasis in PC (Ito et al., 2004). The Warburg effect which means augmented glucose consumption in cancer cells was shown in solid tumors such as pancreatic cancers (Mueckler, 1994). HIF-1α induces the glucose transporter GLUT-1 expression under hypoxic conditions (Chen et al., 2001). The correlation between HIF-1α and GLUT-1 in inducing cancer development suggests GLUT-1 as a potential cancer therapy. It was revealed that glucose transporter GLUT-1 is blocked by apigenin (0–100 μM) under normoxic conditions (Melstrom et al., 2008).

In one study, the effect of apigenin on hypoxia responsive genes in pancreatic cancer was performed by Melstrom et al. (153). The expression level of the VEGF, HIF-1α, and GLUT-1 was examined in S2-013 human PC cells and CD18 cells which were treated with apigenin (0–50 μM) by an enzyme-linked immunosorbent assay (ELISA), Western blot analysis, and real-time RT-PCR in both normoxic and hypoxic conditions. GLUT-1 expression undergoes up-regulation in PC cells in comparison to adjacent controls (*P* < 0.001). Expression of the VEGF, HIF-1α, and GLUT-1 protein is stimulated in S2-013 PC cells and CD18+ cells under hypoxic conditions. Hypoxia-induced up-regulation of these three proteins is inhibited by apigenin (50 μM). Furthermore, apigenin obstructed the expression of the GLUT-1 and VEGF mRNA under hypoxia conditions in the mentioned cell lines. In normoxic and hypoxic conditions, GLUT-1, HIF-1α, and VEGF mRNA transcription and protein production both are suppressed by apigenin. This suggests apigenin as a potential anti-cancer drug for the treatment of the PC (Melstrom et al., 2011). Table 2 illustrates the therapeutic effects of apigenin on PC cells.

**Table 2:** The therapeutic effects of apigenin on pancreatic cancer.

**Dose (s)**	**Target gene (s)**	**Model**	**Type of cell line**	**Effect (s)**	**References**
0 to 100 μM	GLUT-1	*In vitro*	CD18, S2-013	Reduces glucose uptake	Melstrom et al., 2008
50 μM	Cdc6, Cdt1, and MCM7	*In vitro*	CD18, S2013	Anti-tumor effects	Salabat et al., 2008
6 to 50 μM	P53	*In vitro*	Panc1, PaCa44	Overcomes the chemo-resistance	Gilardini Montani et al., 2019
25 mg/kg	Ikaros/ CK2α protein	*In vitro, in vivo*	Panc02	Anti-tumor effects	Nelson et al., 2017
50 μg/kg	Extracellular matrix proteins collagen 1A1 and fibronectin, transforming growth factor-beta, and interleukin-6	*In vitro, in vivo*	PSCs	Anti-tumor effects	Mrazek et al., 2015
0 to 80 μm	Caspase-3	*In vitro*	AsPc-1, Panc-1, MiaPaCa-2	Deceases the cancer cell growth, Induces apoptosis	Wu et al., 2014
23 and 12 μM; 71 and 41 μM	Glycogen synthase kinase-3β/nuclear factor kappa B	*In vitro*	BxPC-3, PANC-1	Anti-tumor effects	Johnson and Gonzalez de Mejia, 2013
0 to 50 μM	nuclear GSK-3β and NF-κB, p65	*In vitro*	BxPC-3	Induces apoptosis, Increases anti-proliferative effects	Johnson and Gonzalez de Mejia, 2013
50 μmol/L	β-AR	*In vitro*	BxPC-3 and MIA PaCa-2	Anti-tumor effects	Pham et al., 2012
1 to 100 μM	Bcl-XL, PUMA, and p53	*In vitro*	BxPC-3, MiaPaCa-2	Anti-tumor effects, Induces apoptosis	King et al., 2012
0 to 50 μM	HIF-1α, GLUT-1, and VEGF	*In vitro*	CD18 and S2-013	Decreases angiogenesis, and glucose uptake	Melstrom et al., 2011
25 μM	pAkt and NF-JB	*In vitro*	CD18 and AsPC-1	Inhibits cell proliferation	Strouch et al., 2009
6.25 to 100 μM	cyclin A, cyclin B, phosphorylated	*In vitro*	cdc2 and cdc25	Inhibits cell growth	Ujiki et al., 2006
0.1 to 10 μM	NAG-1 and p53	*In vivo*	HCT-116 cells	Decreases cell growth	Yang et al., 2014

### Chemotherapy

**Figure 8:**
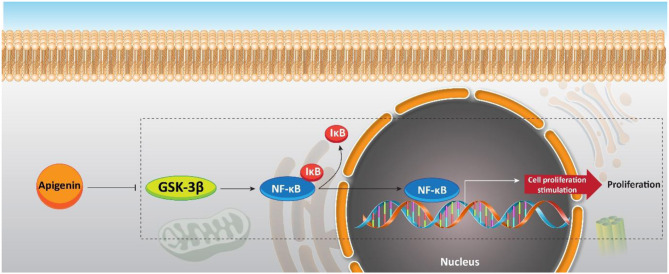
Effect of apigenin on NF-κB signaling, and its upstream mediator GSK-3β in sensitizing pancreatic cancer cells to chemotherapy.

Although a previous study offers an interesting strategy to inhibit the resistance of PC cells with apigenin, increasing evidence demonstrates that apigenin can be applied as a chemosensitizer. Naturally occurring dietary compounds were highlighted due to their positive effects on overcoming the resistance of tumor cells to apoptosis (Johnson and de Mejia, 2011). The natural flavonoid apigenin is a potential molecule for overcoming chemoresistance in pancreatic cancer (Johnson and de Mejia, 2013). Serine/threonine kinase glycogen synthase kinase-3β (GSK-3β) as a potential target for flavonoids is responsible for major upstream regulator of the NF-κB transcriptional activity. NF-κB enters the nuclei of the pancreatic cancer cells (Ougolkov et al., 2005). Conventional chemotherapeutic agents refuse apoptosis by stimulating the NF-κB pathway (Long et al., 2011). GSK-3β activity is inhibited by apigenin (Johnson et al., 2011). Furthermore, this flavonoid has an important role in suppressing pancreatic cancer cell proliferation *in vitro*. Apigenin (0, 10, 25, and 50 μM) activates apoptosis in pancreatic cancer cells by suppressing the GSK-3β/NF-κB signaling pathway (Figure 8) (Johnson and de Mejia, 2013).

**Figure 9:**
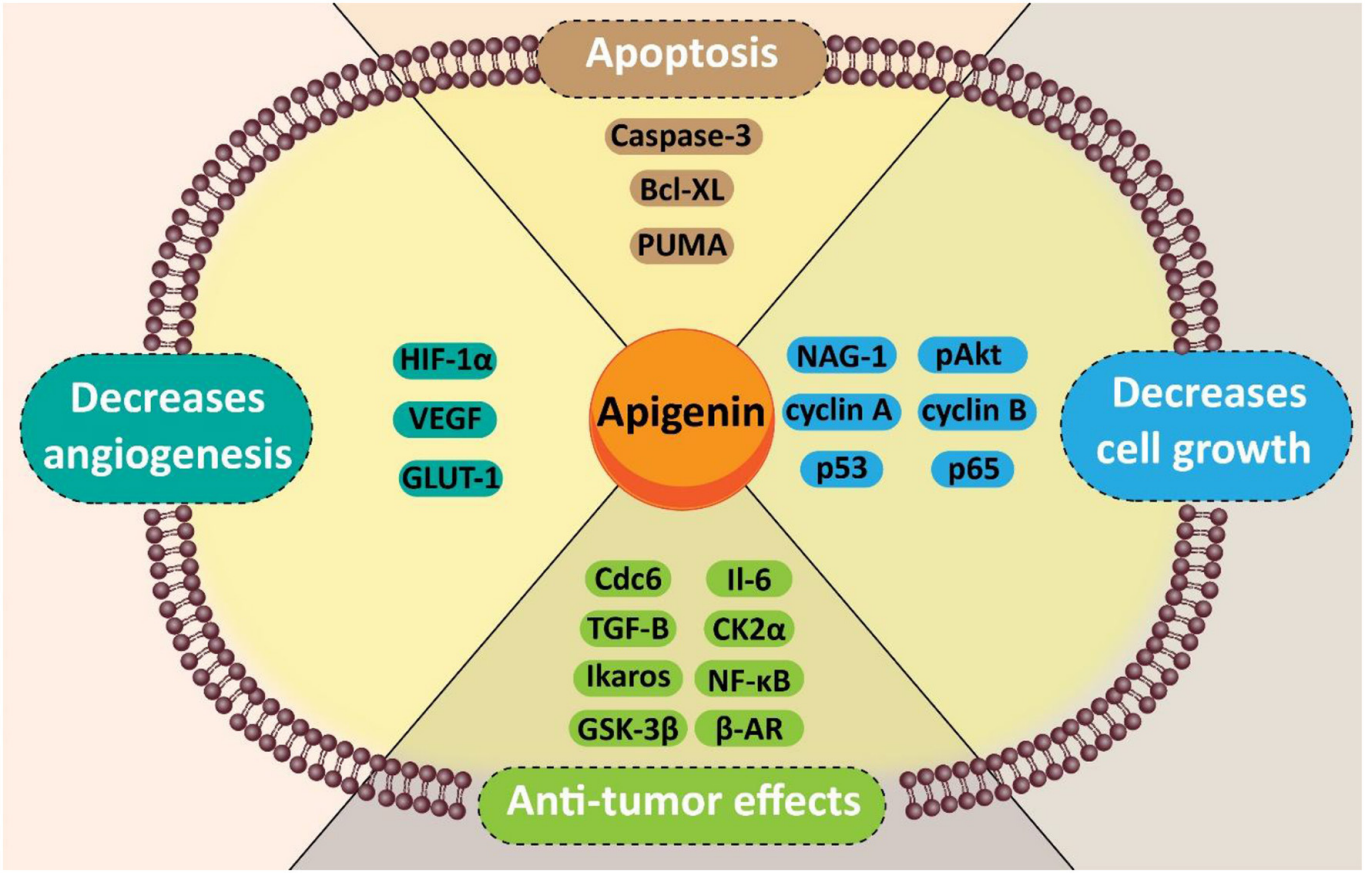
An overview of molecular pathways targeted by apigenin in pancreatic cancer therapy.

According to the experiment conducted by Johnson et al. the ability of the flavonoid apigenin to assist chemotherapeutic drugs in inhibiting the proliferation of BxPC-3 pancreatic cells was examined (Johnson and Gonzalez de Mejia, 2013). An MTS cell proliferation assay with different concentrations of the chemotherapeutic drugs (0–50 μM) and concurrent pretreatment or treatment of flavonoids (0, 6, 24, and 42 h) were conducted. Simultaneous treatment through the chemotherapeutic drugs 5-fluorouracil (5-FU, 50 μM), flavonoid (13, 25, or 50 μM), or gemcitabine (Gem, 10 μM) for 60 h leads to mostly less-than-additive effects (*p* < 0.05). Pretreatment for 24 h with 13 μM of apigenin, followed by Gem for 36 h was ideal for suppressing the proliferation of the cells. Pretreatment of cells with 11–19 μM of apigenin for 24 h leads to 59–73% growth suppression when followed by Gem (10 μM, 36 h). Pretreatment of BxPC-3 human pancreatic cancer cells by low concentrations of apigenin or Lut assist chemotherapeutic drugs in their anti-proliferative properties (Figure 9) (Johnson and Gonzalez de Mejia, 2013).

## Conclusion and Outlook

Since, there have not yet been any reports of apigenin having unfavorable metabolic reactions, involving it in the diet is recommended. Bioactive compounds such as apigenin take part in different metabolic pathways to exert their healing effects. The pharmacokinetic behavior of these bioactive compounds affects their bioactivity as well as tissue distribution. Naturally, apigenin occurs in dimeric forms linked through C–C or C–O–C bonds.

Various pharmacokinetic behaviors and healing effects have been found in flavonoid aglycones and their glycosides. So, C-glycosylation or O-glycosylation of apigenin may have effects on its metabolism, and in turn, show impacts on both its anti-oxidant potential and biological benefits. The decreased anti-oxidant potential of apigenin via O-glycosylation has been demonstrated in an *in vitro* assay (Cai et al., 2006). In another study, Angelino et al. investigated the bioavailability of the apigenin-C-glycosides. They reported no alteration in the absorption of vitexin-2-O-xyloside (VOX) that is an apigenin-8-C-glucoside in a rat model (Angelino et al., 2013). Besides being hydrolyzed to the mono-glycoside, reduced, and conjugated to make a bioavailable glucuronide, VOX also undertakes enterohepatic recirculation. Over the last few years, many studies pointed out the various pharmacological activities and nutraceutical potential of apigenin. For instance, its anti-oxidant properties are well-recognized and apigenin is also considered as a therapeutic agent for different conditions such as autoimmune disease, neurodegenerative disease, inflammation as well as some types of cancers (Salehi et al., 2019). In suppressing the proliferation of cancer cells, apigenin can induce apoptotic cell death *via* increasing ROS generation, the down-regulation of anti-apoptotic factors Bcl-2 and Bcl-xl as well as the up-regulation of apoptotic factors Bax and Bim. Besides, apigenin can induce cell cycle arrest at the G2/M and S phases. In suppressing metastasis of cancer cells, apigenin administration interferes with the PI3K/Akt/mTOR signaling pathway as well as the expression of MMP-9, as a factor involved in the progression and invasion of cancer cells.

In comparison to the other structurally related flavonoids, apigenin showed reduced intrinsic toxicity on normal cells. Despite its importance and useful effects, there is not enough literature on apigenin's beneficial health potential for humans. A good reason may be low solubility of apigenin in water (1.35 μg/mL) and its high permeability (Zhang et al., 2012). These may hamper the *in vivo* studies into apigenin. There are various strategies suggested to increase solubility, such as several delivery systems (nanosuspension, polymeric micelles, liposomes). These approaches, for example, show how solid dispersion could improve the low solubility of therapeutic agents. Furthermore, several injectable nanosized drug delivery systems have been devised, demonstrating that nanocapsules may be a good tactic to lengthen the pharmacological activity of apigenin. Indeed, high metabolic transformation and low bioavailability of some food components have been left as unsolved issues.

It was revealed that the administration of apigenin is beneficial in enhancing the sensitivity of PC cells to chemotherapy. Besides, apigenin affects molecular pathways such as HIF, GLUT-1, and VEGF to disrupt the proliferation and malignant behavior of PC cells. Also, additional molecular pathways and mechanisms (e.g., Wnt, miRs, lncRNAs, epithelial-to-mesenchymal transition) can be considered as down-stream targets of apigenin, and can be discussed in future studies. According to the information presented in clinicaltrials.gov, today just one study has examined the anti-tumor activity of apigenin in clinical trials. Its status is suspended (NCT00609310) and it was supposed to evaluate effect of apigenin on the recurrence of colorectal cancer cells. However, we are still at the beginning stage and there will be more studies in the future to investigate the anti-tumor activity of apigenin in clinical trials.

## Author Contributions

HM was involved in the conception, design, statistical analysis, drafting of the manuscript, and supervised the study. MB, MA, ZB, KI, AZ, PM, HK, MD, and SM contributed to data collection and manuscript drafting. All authors approved the final version for submission.

## Conflict of Interest

The authors declare that the research was conducted in the absence of any commercial or financial relationships that could be construed as a potential conflict of interest.
